# Genetic Landscape and Emerging Therapies in Uveal Melanoma

**DOI:** 10.3390/cancers13215503

**Published:** 2021-11-02

**Authors:** Rino S. Seedor, Marlana Orloff, Takami Sato

**Affiliations:** Department of Medical Oncology, Sidney Kimmel Cancer Center, Thomas Jefferson University, Philadelphia, PA 19107, USA; Marlana.Orloff@jefferson.edu (M.O.); Takami.Sato@jefferson.edu (T.S.)

**Keywords:** uveal melanoma, metastatic uveal melanoma, genetic landscape, targeted therapy, treatment strategy

## Abstract

**Simple Summary:**

Uveal melanoma is the most common primary intraocular malignancy in adults. Unfortunately, metastasis develops in up to 50% of cases and outcomes are poor for these patients. In this review, we discuss our current understanding of the unique genetic landscape of uveal melanoma, and the prognostic and potential therapeutic insight it can provide. By obtaining information on molecular and cytogenetic mutations, patients and their providers can gain important knowledge that may help with surveillance and treatment decisions, including clinical trial enrollment.

**Abstract:**

Despite successful treatment of primary uveal melanoma, up to 50% of patients will develop systemic metastasis. Metastatic disease portends a poor outcome, and no adjuvant or metastatic therapy has been FDA approved. The genetic landscape of uveal melanoma is unique, providing prognostic and potentially therapeutic insight. In this review, we discuss our current understanding of the molecular and cytogenetic mutations in uveal melanoma, and the importance of obtaining such information. Most of our knowledge is based on primary uveal melanoma and a better understanding of the mutational landscape in metastatic uveal melanoma is needed. Clinical trials targeting certain mutations such as *GNAQ*/*GNA11*, *BAP1*, and *SF3B1* are ongoing and promising. We also discuss the role of liquid biopsies in uveal melanoma in this review.

## 1. Introduction

Uveal melanoma (UM) is the most common primary intraocular malignancy in adults, with an estimated incidence of 5.2 per million population in the United States [1,2,3]. Up to 50% of UM patients will develop systemic metastasis despite successful treatment of the primary tumor, most commonly in the liver [4,5]. Overall metastatic disease portends a poor prognosis, with an estimated one-year survival of 15 to 67% and median overall survival ranges from 4 to 19 months [5,6,7,8,9,10,11,12,13,14,15,16]. 

Currently, there is no treatment for metastatic uveal melanoma (MUM) approved by the United States Food and Drug Administration. A recent systemic review of PubMed studies over the past 40 years demonstrated no statistically significant difference in overall survival between different treatment modalities for MUM; local therapies including intra-arterial liver chemotherapy, isolated liver perfusion, and selective internal radiation therapy showed some advantage, although not statistically significant, in comparison to systemic therapies (chemotherapy, immunotherapy, immunosuppression, targeted therapy) [17]. Immunotherapy has dramatically improved outcomes for cutaneous melanoma patients, but a similar clinical benefit has not been seen in MUM [8]. Retrospective studies of immunotherapy in MUM patients reported low objective response rates ranging from 10–21% with combination ipilimumab and nivolumab, 3.6% with anti- programmed cell death 1 (PD-1) antibodies, and 5% with ipilimumab monotherapy [8,18,19,20,21,22]. Two recent prospective phase II studies of combination ipilimumab and nivolumab in MUM patients showed a median progression-free survival (PFS) of 3 and 5.5 months, median overall survival (OS) of 12.7 and 19.1 months, and overall response rate (ORR) of 11.5% and 18% [14,23]. This is disappointing in comparison to the CHECKMATE067 study of combination ipilimumab and nivolumab in metastatic cutaneous melanoma patients achieving an impressive median OS of 72.1 months and ORR of 58% [24,25]. To improve immune response against metastatic uveal melanoma, various loco-regional approaches have been combined with systemic immune checkpoint inhibitors. There are many clinical trials that are ongoing that combine immunotherapy with other therapies in MUM in hopes of improving outcomes: isolated hepatic perfusion with ipilimumab and nivolumab (NCT04463368), percutaneous hepatic perfusion with ipilimumab and nivolumab (NCT04283890), arginine deprivation with ipilimumab and nivolumab (NCT03922880), radioembolization with Yttrium90 with ipilimumab and nivolumab (NCT02913417), immunoembolization with ipilimumab and nivolumab (NCT03472586), stereotactic radiosurgery with pembrolizumab (NCT02858869), entinostat with pembrolizumab (NCT02697630), and autologous CD8+ SLC45A2-specific T lymphocytes with cyclophosphamide, IL-2, and ipilimumab (NCT03068624). 

The genetic landscape of cutaneous melanoma (CM) appears to be quite different from uveal melanoma. The Cancer Genome Atlas (TCGA) has divided cutaneous melanoma into the four genomic subtypes of *BRAF*, *RAS* (*N*/*H*/*K*), *NF1*, and Triple-WT (wild-type) [26]. These subtypes are based on the presence of *BRAF*, *NRAS*, and *NF1* mutations in 52%, 28%, and 14%, respectively. With the exception of the Triple-WT subtype, a UV signature was noted in greater than 90% of samples. The tumor mutational burden was high in CM at 18 per Mb [26]. 

**Figure 1:**
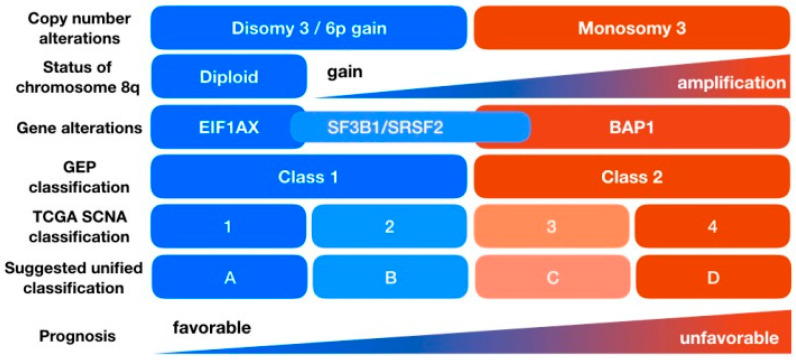
Schema of TCGA UM Subtypes. Reprinted from “Molecular Characteristics of Uveal Melanoma: Insights from the Cancer Genome Atlas (TCGA) Project,” by Matheiu F. Bakhoum and Bita Esmaeli. 2019, Copyright 2019 by authors [28].

In contrast, the TCGA analysis on 80 primary UM has divided uveal melanoma into four different subtypes: two associated with poor-prognosis monosomy 3 and two with better-prognosis disomy 3 (Figure 1) [27,28]. Cluster 1 and 2 retained both copies of chromosome 3 and were enriched for 6p gain. Clusters 3 and 4 UMs were associated with monosomy 3. Gain of 8q was present in clusters 2, 3, and 4, with higher copy gains or amplifications in the higher number clusters. *GNAQ*, *GNA11*, *BAP1*, *SF3B1*, *EIF1AX*, *CYSLTR2*, *SRSF2*, *MAPKAPK5*, and *PLCB4* were the nine genes found to be significantly mutated in primary UM. There was no UV radiation mutational signature and mutational burden was low at 1.1 per Mb [27].

Similar to the TCGA analysis, other large genomic analyses of UM have also categorized UM tumors into four categories based on recurrent chromosomal aberrations and somatic mutations [27,29,30,31]. Interestingly the genomic analysis by Johansson et al. found all of their iris tumors to have genomic features associated with ultraviolet radiation damage [29]. This may be explained by the anterior position of the iris, resulting in direct exposure to sunlight that breaches the cornea. Another outlier is methyl-CpG-binding domain 4 (MBD4) gene mutations, both germline and somatic loss of function variants, which lead to increased mutational burden and a better response to immunotherapy [32,33,34]. Overall though, compared to many other tumor types UM exhibit a relatively low degree of genomic instability and aneuploidy [35].

It is important to note that the DNA-based and RNA-based tests (including the test by Castle Biosciences) commonly utilized by ophthalmologists and oncologists analyze the primary eye specimen [27]. Genomic analyses of UM have been primarily on primary UM samples [29,30,31]. Analyses on metastatic UM have been small and few in number. Karlsson et al. specifically performed whole-genome sequencing (WGS) of 32 metastatic UM (6 subcutaneous and 26 liver) and found similar somatic mutations but more frequent broad copy number events compared to primary tumors from TCGA [36]. This included loss of chromosome 3, 6q, and 17p, as well as gains of 5p and 8q. Two of their tumors also had *CDKN2A* deletions which are rarely seen in primary UM [36]. Likewise, genomic analyses that compared primary to metastatic UM noted enrichment of most somatic alterations, mainly in copy number changes of 6q (loss), 1q (gain), and 8q (gain) [33,37]. Loss of heterozygosity on chromosome 3 was never acquired during tumor progression or in metastases [33]. Unlike most other tumor types, UMs continues to genetically evolve as they progress from primary to metastatic disease, gaining more oncogenic mutations along the way [37,38,39]. When compared to liver metastases from CM, the genomic profiles seen in liver metastases from UM were very different [40]. Tumor mutational burden was low in liver UM compared to that of CM. MelanA expression was higher in UM compared to CM, but PD-L1 expression was lower (<1% in 93% of the UM samples vs 73% in CM samples). While the extent of immune infiltration was similar for CM and UM liver metastases, the ratios of exhausted CD8+ T cells to cytotoxic T cells, to total CD8+ cells and to Th1 cells, were significantly higher in UM [40]. More comprehensive genomic studies strictly on metastatic specimens are needed, as it may help lead to a better understanding of differences in disease course and treatment responses.

In this review we will focus on the current understanding of the molecular and cytogenetic mutations in uveal melanoma (largely based on primary UM), and how this information can provide valuable insight into a patient’s prognosis, surveillance, and treatment options. We will also discuss the potential role of liquid biopsies in UM.

## 2. Genetic Landscape

### 2.1. Chrosome Copy Number Aberrations

Primary UM are characterized by recurrent chromosome aberrations in chromosomes 1, 3, 6, 8, 9, and 16. These cytogenetic alterations are tightly linked to prognosis, and are used for stratification of patients into risk categories. In primary UM, the most common chromosomal aberrations are 1p loss (28–34%), 1q gain (24%), 3 loss (50–61%), 6p gain (28–54%), 6q gain (28–54%), 6q loss (35–37%), 8p loss (17–28%), 8q gain (36–63%), 9p loss (24%), and 16q loss (16%) [41,42,43]. In particular, monosomy 3 and gain of chromosome 8q correlate with increased metastatic risk, with increasing percentages of monosomy 3 and gain of 8q in tumor cells showing a correlation with worse prognosis [44]. 8p loss has also been associated with a more rapid onset of metastasis, indicative of metastatic efficiency [35]. On the contrary, chromosome 6p gain correlated with a good prognosis, suggesting this aberration could have a functionally protective effect [45]. It may also be that 6p gain portends a better prognosis simply because it is mutually exclusive with monosomy 3 [35]. Monosomy 3 appears to be an early event, while loss of 1p, 8p, and gain of 8q as secondary events in UM development, relating to large tumor size [42,43]. 

Of note, chromosome 3 loss uncovers recessive BRCA 1-associated protein (*BAP1*) mutations, which is located on chromosome 3p21.1 [46]. *BAP1* encodes a deubiquinating enzyme (nuclear ubiquitin carboxy-terminal hydrolase) and is a known tumor suppressor gene. Inactivation of *BAP1* has been observed in 47% of primary UM and 91% of metastatic UM, correlating with the understanding that loss of *BAP1* is a key event to metastasis [36,47]. Deletion of chromosome 3 also usually accompanies the development of the class 2 gene expression signature, suggesting that one or more genes on chromosome 3 may regulate the emergence of a cancer stem-like phenotype [46]. The 3-year relapse free survival rate for monosomy 3 tumors is 50% [48].

The *MBD4* gene encodes a DNA glycosylate and is another tumor suppressor gene located on chromosome 3 [49]. Germline *MBD4* mutations have been found to predispose to UM (9.15-fold increase in comparison to the general population), and have also been rarely reported in gastrointestinal malignancies, central nervous system malignancies, and acute myeloid leukemia [49,50]. The prevalence of *MBD4* germline mutations in UM is approximately 0.23–0.7% [34,49]. 

Although MUM tumors typically have a low response rate to immunotherapy, there have been two case reports of success with immunotherapy with germline *MBD4* mutated tumors [32,50]. *MBD4* mutations result in a CpG > TpG hypermutator phenotype with a high tumor mutation burden, which may explain the immunotherapy responsiveness [31,49]. Clinical trials using immunotherapy in MUM patients may want to consider stratifying for *MBD4* mutations given the high tumor mutation burden of these tumors and likelihood of response to immune checkpoint inhibitors. 

### 2.2. Gene Expression Profiling (GEP)

Aside from cytogenetic status as a way to classify tumors, primary UM is often also classified by gene expression profiling, which relies on a comparison of relative levels of RNA from a 15-gene panel. The commercially available Castle Biosciences DecisionDx-UM Gene Expression Profile classifies tumors into Class 1A and 1B which are low risk, and Class 2 which are high risk of metastasis [51]. The assay comprises 12 discriminating genes (*CDH1*, *ECM1*, *EIF1B*, *FXR1*, *HTR2B*, *ID2*, *LMCD1*, *LTA4H*, *MTUS1*, *RAB31*, *ROBO1*, and *SATB1*) and 3 control genes (*MRPS21*, *RBM23*, and *SAP130*) performed on a microfluidics platform [52]. Class 1A tumors are classified as “very low risk,” with a 2% risk of metastasis within 5 years. Class 1B tumors are classified as “low risk,” with a 21% risk of metastasis within 5 years. Class 2 tumors are classified as “high risk,” with a 72% risk of metastasis within 5 years. Prognostic validation was performed through a prospective study by the Collaborative Ocular Oncology Group [53]. The authors noted the assay may be superior to chromosome 3 status for clinical prognostic testing [53]. More recently, the 5-year outcome results of a prospective study of 89 patients enrolled at four centers showed 5-year Class 1 and 2 metastasis rates to be 10% and 58%, respectively. Metastasis-free survival rates for Class 1 and 2 tumors were 90% and 41%, respectively [54].

The GEP of Class 1 tumors closely resemble normal uveal melanocytes and low-grade uveal melanocytic tumors, while the GEP of Class 2 tumors on the other hand resemble the transcriptome of primitive neural/ectodermal cells [55,56]. Class 1 tumors have been shown to harbor mutations in the translation elongation factor *EIF1AX* and the splicing factor *SF3B1*, whereas mutations in the tumor suppressor gene *BAP1* are strongly associated with Class 2 tumors [57]. There is a significant association between class 1 and disomy 3, and between class 2 and monosomy 3; however, the GEP and chromosome 3 results were discordant in 20.8% of cases [53]. 

More recently the messenger-RNA expression of cancer-testis antigen PRAME (Preferentially Expressed Antigen in Melanoma) is being tested along with GEP. The expression of PRAME is believed to represent an independent biomarker providing an additional layer of prognostic precision to the Class 1/Class 2 GEP system [57].

### 2.3. Somatic Mutations

#### 2.3.1. GNAQ and GNA11

Mutually exclusive somatic mutations in the G-protein pathway-associated *GNAQ* and/or *GNA11* (92.5%), *CYSTLR2* (4%), or *PLCB4* (2.5%) genes are found in uveal melanoma [27]. In primary UM *GNAQ* and *GNA11* mutations occur in 50% and 45%, respectively [28]. Mutations in *GNAQ* primarily affect either the amino acid Q209P/L (90%), R183Q (5%), or G48*/V (5%). Similarly, mutations in *GNA11* primarily affect either the amino acid Q209L (94%), R183C (3%) or R166H (3%) [28,58]. These mutations lead to the constitutive activation of the Gαq and Gα11 subunits by abrogating their intrinsic GTPase activity required to return them to an inactive state, which results in the constitutive activation of G-protein coupled signaling pathways such as MAPK, PI3K, PKC, Akt/mTOR, Rac/Rho, Wnt/β-catenin, and Hippo [58,59,60,61,62]. *GNAQ*/*11* mutations are also found in benign uveal nevi, rarely in cutaneous or conjunctival melanoma [59]. They are seen in most UM regardless of cytogenetic status, GEP class, or *BAP1* status [63,64]. *GNAQ*/*11* mutations are felt to occur early in UM as tumor-initiating mutations (precursor events) [35]. A ‘second hit’ event then leads to malignant transformation. *GNAQ*/*11* mutations do not appear to be prognostic [35,59,63]. Limited data suggests no statistically different survival between *GNAQ* and *GNA11* and the type and location of mutations, although there may be a trend toward increased survival among patients with a mutation in Q209P, compared to Q209L mutation [58,65,66].

#### 2.3.2. EIF1AX, SF3B1, SRSF2, and BAP1

Mutually exclusive mutations in *EIF1AX* (13%), *SF3B1* (23%), *SRSF2* (4%), and *BAP1* (33%) are the second oncogenic event of UM and are associated with markedly distinct prognoses [28]. Tumors with mutations in *EIF1AX* all have disomy of chromosome 3 and appear to have the lowest risk of metastasis [27,62,67,68]. *SF3B1* and *SRSF2* mutations also mostly have disomy of chromosome 3 (88%) and develop metastases late (median 8.2 years after initial diagnosis) [28,62,68,69]. *BAP1* mutations are all associated with monosomy 3 and early metastatic risk [28].

The *EIF1AX* gene encodes for the eukaryotic translation initiation factor 1A, an x-linked protein that plays a role in the initiation of translation of mRNA to protein through the recruitment of the ternary complex and assembling of the 43S preinitiation complex (PIC) [62]. *EIF1AX* mutations are also found in papillary thyroid carcinomas and ovarian carcinomas, with co-occurrences and cooperation with *RAS* mutations [27,62,70,71]. Coexpression of mutant *NRAS* and *EIF1AX* proteins in low-grade ovarian carcinoma cells promoted proliferation and clonogenic survival [70]. Similarly, cooperation of *RAS* and *EIF1AX* mutations were demonstrated in papillary thyroid cell lines and mouse models [71].

The splicing factor gene mutations *SF3B1* (splicing factor B3 subunit 1) and *SRSF2* (Serine And Arginine Rich Splicing Factor 2) affect proteins involved in 3′ splice site recognition [62]. *SRSF2* specifically encodes serine/arginine-rich proteins that bind exonic splicing enhancers, resulting in misregulated exon inclusions that causes an aberrant splicing pattern of many genes including the tumor suppressor genes *ARMC10* and *EZH2*. *SRSF2* mutations are also commonly found in chronic myelomonocytic leukemia (47%) and myelodysplastic syndrome (15%) [62]. *SF3B1* is involved in the recognition of the branch point sequence by encoding the U2 small nuclear riboprotein complex (U2-snRNP) [62]. *SF3B1* mutations result in an aberrant splicing pattern of important apoptotic genes such as MCL1 and BCL2/xL through the usage of an alternative 3′ splice site upstream the canonical 3′ splice site [62]. *SF3B1* mutations are found in hematologic malignancies such as myelodysplastic syndrome (MDS), myeloproliferative neoplasms, chronic myeloid leukemia, and acute myeloid leukemia (AML), as well as solid tumors such as uveal melanoma, mucosal melanoma, leptomeningeal melanoma, blue nevus-like cutaneous melanoma, neuroblastomas that arise following chromothripsis, estrogen receptor-positive breast cancer, pancreatic ductal adenocarcinoma, prostate cancer, and prolactinoma [72]. 

*BAP1*, or BRCA1-associated protein 1, encodes a nuclear deubiquinating enzyme called ubiquitin carboxy-terminal hydrolase (UCH) [46]. In addition to the UCH catalytic domain, BAP1 contains a UCH37-like domain, binding domains for BRCA1 and BARD1, and a binding domain for HCFC1. BAP1 forms a tumor suppressor heterodimeric complex with BRCA1 and BARD1 that is involved in cell proliferation, DNA damage response, and differentiation processes through influencing chromatin remodeling [46,73]. The binding of BAP1 with HCFC1 interacts with histone-modifying complexes [46]. BAP1 also forms the Polycomb group repressive deubiquitinase complex with ASXL1 which is involved in stem cell pluripotency and other developmental processes [46]. 

Most *BAP1* mutations in UM are somatic, with only 1–2% with germline *BAP1* mutations [74,75,76,77]. Germline mutations in *BAP1* are inherited in an autosomal dominant pattern, with the inheritance of a non-functional *BAP1* allele with the remaining allele inactivated later in life (two-hit hypothesis) [78]. *BAP1* mutation penetrance is relatively high, with one type of cancer developing in more than 80% of gene carriers [79]. *BAP1* tumor predisposition syndrome is associated with UM, malignant mesothelioma, cutaneous melanoma, renal cell carcinoma, basal cell carcinoma, hepatocellular carcinoma, cholangiocarcinoma, and meningioma [80]. The point prevalence of uveal melanoma development in germline *BAP1* patients is reported to be about 2.8% at a median age of 50.5 years [81]. When *BAP1* carriers develop UM, the tumor tends to be larger in diameter, involve the ciliary body, and develop at a younger age when compared to non-*BAP1* carriers [75]. There is no consensus on surveillance recommendations for patients with germline *BAP1*, but several groups have recommended a multidisciplinary approach beginning at a young, pre-adolescent age and 5 years before the first reported age at diagnosis of the malignancy associated with germline *BAP1* [79,82,83,84,85]. Ophthalmological and dermatologic exams should be performed routinely. Malignant mesothelioma and renal cell carcinoma screening recommendations are variable, but involve physical examination and imaging (CT, MRI, ultrasound).

Whether germline *BAP1* results in increased risk of metastasis and poorer outcome in comparison to somatic *BAP1* or mutation-negative tumors is unclear. Germline *BAP1* mutation appeared to result in more metastatic disease compared to non-*BAP1* carriers (71.4% vs. 18.0%) in a small comparison between 7 germline *BAP1* UM and 455 UM without germline *BAP1* [75]. In another small study, germline *BAP1* was detected in 4 of 50 metastatic UM cases and 0 of 50 nonmetastatic UM cases (8% vs. 0%, *p* = 0.059), implying a small but significant increase in metastatic risk [86]. However, a larger cohort of UM tumors carrying somatic *BAP1* mutations (*n* = 43), germline *BAP1* mutations (*n* = 11), and no mutations (*n* = 88), showed an increased frequency of metastasis in somatic but not germline when compared to mutation-negative [87]. Similarly, DNA extracted from 142 UM tumors showed somatic *BAP1* portended a shorter time to metastasis and poorer metastatic outcome compared to germline or mutation-negative tumors; this was not seen between germline and mutation-negative tumors [87]. Rare genetic syndromes such as *BAP1* tumor predisposition syndrome are at risk of ascertainment bias, where unaffected carriers or carriers with unusual clinical presentation are less likely to be captured. This may explain the differences in metastatic potential in the studies [88]. The small number of germline tumors in the studies due to the rarity of germline *BAP1* is also a limitation. At this time it is unclear as to how germline *BAP1* patients with UM should be monitored.

## 3. Surveillance

Clinical factors predictive of metastasis have been evaluated in several large retrospective reviews [89,90,91]. Increased metastatic risk was correlated with older patient age, ciliary body location, increasing tumor diameter, increasing tumor thickness, darkly pigmented tumor, and the presence of subretinal fluid, intraocular hemorrhage, or extraocular extension [89]. There was also a 2-fold increase in risk for metastasis and death with each increasing tumor size category in the American Joint Committee on Cancer 7th edition [90]. Epithelioid cell type, higher values of mean diameter of ten largest nucleoli, higher microvascular density, extravascular matrix patterns, high mitotic activity, tumor-infiltrating lymphocytes, tumor-infiltrating macrophages, higher expression of insulin-like growth factor-1 receptor, higher expression of human leukocyte antigen Class I and II, MET and PRAME expression are histopathologic features suggestive of poor prognosis [57,91,92]. Additionally, based on the prognostic significance of chromosome copy number aberrations, gene expression profiling, and somatic mutations in primary UM tumors, recommendations for surveillance can be tailored to a patient.

Currently, the NCCN guidelines divide primary UM patients into three risk categories [93]. Patients are considered low risk if their primary UM is GEP class 1A, has disomy of chromosomes 3 or gain of chromosome 6p, harbors a *EIF1AX* mutation, or is T1 based on AJCC guidelines. Patients are considered medium risk if their tumor is GEP class 1B, T2 or T3 based on AJCC guidelines, or harbors a *SF3B1* mutation. Finally, patients are considered high risk if their tumor is GEP class 2, has monosomy 3 or gain of chromosome 8q, harbors a *BAP1* mutation, has PRAME expression, or is T4 based on AJCC guidelines. Risk stratification to determine the frequency of follow-up should be based on the highest risk factor present [93]. 

Based on these three risk categories, the NCCN guidelines recommends surveillance imaging every 12 months for low risk, every 6 to 12 months for 10 years for medium risk, and every 3 to 6 months for 5 years then every 6 to 12 months for 5 more years for high risk patients [93]. Recommended surveillance imaging of the liver includes contrast-enhanced MR or ultrasound, with modality preference determined by expertise at the treating institution. Additionally, contrast-enhanced CT of the chest, abdomen, and pelvis or dual energy subtraction chest x-ray may also be performed. The NCCN guidelines recognize that some patients may elect to forgo surveillance imaging after discussions with their treating physicians, as there are limited options for systemic recurrence, and that regular imaging may cause patient anxiety [93].

We take a stronger stance than the NCCN guidelines and highly recommend patients undergo molecular and cytogenetic testing for risk stratification, and as we later discuss, for treatment consideration. We also strongly recommend patients pursue surveillance imaging with MRI of the abdomen and chest imaging (CT or x-ray) based on risk instead of watchful waiting. MRI of the abdomen detects small live metastases missed on PET, avoids the cumulative radiation risk of whole-body CT and PET, and obtained every six months has been shown to detect the metastases before the onset of symptom in 92% of cases [94,95,96]. Identifying metastatic disease earlier may provide more treatment options for patients, as advanced stages can cause symptoms and result in life-threatening liver failure. Improved response rates and survival outcomes have been seen when the tumor burden is lower at the initiation of liver-directed therapies including liver resection [97,98,99,100,101,102,103]. Although we agree that treatment options for metastatic disease are limited, there have been recent advancements in the field with liver-directed therapy, immunotherapy, and newer therapies such as IMCgp100 [13,14,23,104]. Finally, optimizing surveillance recommendations based on individual metastatic risk has the potential to reduce the burden for low-risk patients, through lower healthcare cost, anxiety, and reduced radiation exposure as seen in other cancers [105,106,107]. 

Several studies have shown that physicians utilizing cytogenetic and molecular testing for their UM patients are following surveillance plan recommendations aligned with metastatic risk [54,108,109,110,111]. A multicenter prospective study of 138 UM patients who were tested with the commercially available Castle Biosciences DecisionDx-UM Gene Expression Profile found that Class 2 patients had significantly higher surveillance intensity [110]. Not unexpectedly, medical oncology referrals were also more common for high-risk Class 2 patients, likely for consideration of adjuvant clinical trials [110].

Patients also appear to prefer receiving prognostic information even when there are no prophylactic measures that might improve prognosis. A study of 99 UM patients found that the majority (97%) wanted prognostic information despite being informed that the result would not influence medical care [112]. There were no significant differences between quality of life and depressive symptoms in psychological and physical domains among the groups (monosomy 3, disomy 3, inconclusive). Nearly all patients felt counseling should be offered when receiving the prognostic information. Patients reported wanting to plan for the future as their motivation for having a prognostic test [112]. Similarly, another study of 298 UM patients found that patients want prognostic information as it helped to plan for the future [113]. They also felt they gained a sense of control and hopefulness that continuing medical research and surveillance would prolong their survival, and knowing prognostic information reduced their uncertainty and its accompanying anxiety. None of the patients interviewed in detail expressed any regret about having the prognostic test and there was no evidence of harm [113]. 

## 4. Adjuvant Therapy

Identification of high-risk patients by molecular or cytogenetic testing can also be important for adjuvant therapy consideration. In the past dacarbazine (DTIC), methanol-extraction residue of bacilli Calmette-Guérin, low-dose interferon-alpha, and fotemustine were tested as adjuvant therapy without improvement in survival [47]. At this time there are no adjuvant therapies approved for UM, but there are several trials ongoing for high-risk patients that are promising (Table 1). In particular, there are trials targeting c-Met and c-Kit, which are tyrosine-kinase receptors that activate the RAS/ERK and PI3-kinase pathways [47].

Sunitinib targets VEGF/PDGFR along with C117 (c-Kit), which is vastly expressed in MUM [114]. Sunitinib showed some response in the metastatic setting and is now being studied in the adjuvant setting for high-risk UM patients. In a pilot study of 20 MUM patients, 17 of whom failed previous treatments, one PR and 12 SD was seen with a median PFS and OS of 4.2 and 8.2 months, respectively [114]. The degree of c-Kit expression in MUM cells was not associated with longer PFS or OS [114]. In the adjuvant setting, a retrospective analysis of sunitinib given for 6 months to high-risk UM patients demonstrated longer OS compared to historical controls (HR 0.53, *p* = 0.041) [115]. Adjuvant sunitinib was then compared to 6 months of valproic acid (histone deacetylase inhibitor) in a randomized phase II study, and the 2-year OS rate and 18-month RFS rate were similar between the groups [116]. Currently, there is an actively recruiting randomized phase II trial comparing adjuvant sunitinib with valproic acid for 12 months compared to surveillance in high-risk patients (NCT02068586). 

Crizotinib is a selective small-molecule inhibitor against c-Met, anaplastic lymphoma kinase, and ROS1 [117]. In a UM metastatic mouse model, crizotinib was found to significantly decrease distant metastases compared to untreated controls [117]. A phase II trial of 48 weeks of adjuvant crizotinib in high-risk UM is ongoing (NCT02223819). As of 01/31/2020 the median RFS was 30.6 months and median OS was not reached for 34 patients [118]. 

Adjuvant dendritic cell vaccination is also being studied in UM. A phase II trial of adjuvant DC vaccination in patients with high-risk cytogenetic characteristics was conducted in the Netherlands. Due to the low accrual rates, the trial was stopped prematurely, but of the 23 patients that received at least 1 cycle, median disease-free survival was 34.5 months with a median OS of 51.8 months [119]. A randomized open-label phase III study of adjuvant vaccination with tumor RNA-loaded autologous DCs in patients with resected monosomy 3 UM is open in Germany (NCT01983748). 

Finally, there are several adjuvant immunotherapy trials currently ongoing. There is a phase II single-arm multicenter study of adjuvant ipilimumab combined with nivolumab for high-risk UM patients (NCT03528408). There is also a randomized phase II trial of neoadjuvant and adjuvant nivolumab with or without ipilimumab or relatlimab (anti-LAG-3) in melanoma that is including any risk stage IIIB to stage IV uveal melanoma patients (NCT02519322). Adjuvant ipilimumab alone in 10 high-risk UM patients demonstrated 80% distant disease free survival at 36 months in a phase I/II trial [120]. 

## 5. Treatment Based on Mutational Profile

### 5.1. GNAQ and GNA11

**Figure 2:**
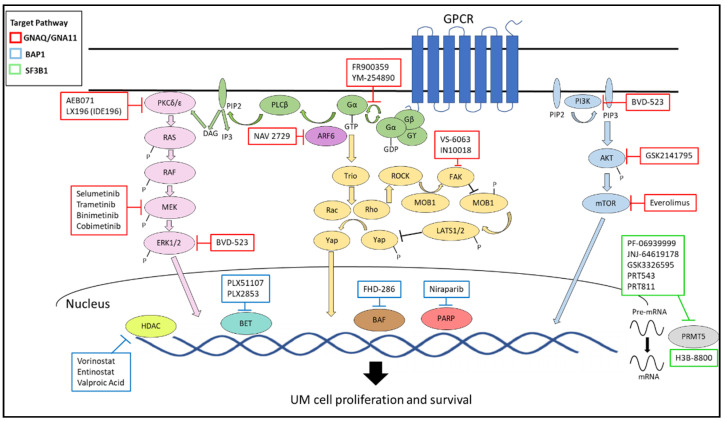
Signaling Pathways in UM. Adapted from “Targeted Therapy of Uveal Melanoma: Recent Failures and New Perspectives” by Michela Croce et al. 846 [121].

Direct targeting of *GNAQ* and *GNA11* may be difficult as these mutations abrogate the intrinsic GTPase activity that would normally allow these proteins to return to their GDP-bound inactive state. The focus, instead, has been to target downstream signaling molecules that are activated by *GNAQ*/*11* mutations such as the RAF/MEK/ERK, PLC¦Á/PKC, PI3K/AKT/mTOR, and Trio/Rho/Rac/YAP1 pathways (Figure 2) [121]. Unfortunately, the results of therapies targeting these pathways have overall been disappointing. Recent reviews by Croce et al. and Mallone et al. summarize the most recent studies of targeted therapies in UM [47,121]. Ongoing targeted therapy trials for MUM are listed in Table 2. 

YM-254890 and FR900359 are two Gαq/11 inhibitors that show promise in vitro, but have not been evaluated for clinical application. YM-254890, a bacterial cyclic depsipeptide, prevents GDP release and selectively inhibits Gαq activation [122]. FR900359 is a plant-based cyclic depsipeptide that acts similarly to YM-254890, selectively inhibiting Gαq/11/14 resulting in cell-cycle arrest and induction of apoptosis [123,124,125]. FR900359 also inhibits ERK1/2 activation, migration and reinstated melanocytic differentiation [123].

ARF6, a small GTPase and ADP-ribosylation factor 6, may be another potentially actionable target for UM, as it is required for oncogenic Gαq signaling and β-catenin signaling [121]. ARF6 facilitates membrane vesicle trafficking of GαQ as well as trafficking of β-catenin to the nucleus, resulting in activation of transcription factors that promote invasion and metastasis [121]. NAV-2729 is an ARF6-specific small molecule inhibitor that has shown the reduction of UM cell proliferation in vitro and in orthotopic xenografts [126]. 

As *GNA1Q*/*11* mutations lead to the constitutive activation of the mitogen-activated protein kinase (MAPK) pathway, downstream effectors have been investigated as targets. Unfortunately, thus far no inhibitor has been successful in UM. A phase II study of an ERK1/2 inhibitor (ulixertinib, BVD-523) failed to demonstrate activity in patients with MUM [127]. There was a systematic review of three open-label phase II, two open-label phase I, and one placebo-controlled phase III trial of different MEK inhibitors in MUM showing a disappointing median PFS of 3.1 weeks to 16 weeks and an overall response rate of 0 to 14% [128]. Despite encouraging results of a phase II study of selumetinib compared with investigator-choice chemotherapy, the phase III study of selumetinib in combination with DTIC (SUMIT trial) failed to show a clinical benefit [129,130,131]. Other trials included in the review were for trametinib monotherapy [132,133], trametinib with uprosertib (AKT inhibitor) [132], and binimetinib with sotrastaurin (PKC inhibitor) (NCT01801358). Mergener et al. demonstrated that *BAP1* mutations and monosomy 3 were associated with higher resistance to MEK inhibition [73]. Their analysis suggested that these tumors with *BAP1* mutation are more resistant to MEK inhibitors because downregulation of eukaryotic translation initiation factor 2A (EIF2A) seen in *BAP1* mutated tumors may lead to a decrease in ribosome biogenesis while inducing an adaptive response to stress [73]. 

There is still active interest in pursuing MEK inhibitors in combination with other therapies for the treatment of MUM. The randomized phase II study of selumetinib alone or in combination with paclitaxel was recently completed (SelPac, ISRCTN29621851). Selumetinib has been shown to enhance paclitaxel-induced tumor apoptosis in preclinical models [134]. The primary analysis presented at ESMO 2019 showed a slight statistical improvement in median PFS but not OS. Median PFS and OS in the combination arm was 4.8 months and 9 months, compared with the monotherapy arm of 3.4 months and 10 months [135]. ORR was 14% in the combination and 4% in the monotherapy arm [135]. The phase IB open label trial of intermittent selumetinib dosing in UM is still recruiting (NCT02768766). Intermittent dosing of selumetinib hopes to test a higher drug dose for more effective blockage of the MAPK pathway, prevent resistance development, and achieve a better toxicity profile. Treatment-related adverse events such as diarrhea and acne-like skin rash were observed in 97% of patients treated with selumetinib in the previous phase II study [130]. Finally, UM patient-derived xenograft models suggest combining selumetinib with an ERK inhibitor or mTORC1/2 inhibitor may be an effective combination [136].

PKC inhibitors are also being investigated as monotherapy or in combination in MUM. *GNAQ*/*11* mutations result in activation of PLCβ which produces diacylglycerol and triggers the activation of the PKC pathway. The pan-PKC inhibitor sotrastaurin (AEB071) as monotherapy showed 1 partial response (PR), 47% disease stabilization (SD), and a PFS of 15.4 weeks [137]. The phase I clinical trial evaluating LXS196, a new generation PKC inhibitor, alone or in combination with HDM201 (MDM2 inhibitor) in MUM is active, but no longer recruiting (NCT02601378). Among the 66 evaluable patients treated with LXS196 monotherapy, 6 had a PR and 45 had SD as their best response, suggesting promising clinical activity for LXS196 as a single agent with manageable toxicity profile [138]. In 2018 Novartis entered into an exclusive license agreement with IDEAYA Biosciences to develop and commercialize LXS196, and the compound was recorded as IDE196 for future development. IDE196 (darovasertib) is currently being tested as monotherapy and in combination with either binimetinib (MEK inhibitor) or crizotinib (MET inhibitor) in an ongoing phase I/II clinical trial of solid tumors harboring a *GNAQ*/*GNA11* mutation (NCT03947385). Based on a preliminary analysis, darovasertib monotherapy resulted in a 57% 1-year overall survival and 13.2 month median OS in a predominantly 2nd and 3rd line therapy MUM patient population [139]. The combination of darovasertib and binimetinib resulted in tumor reduction in 79% of evaluable MUM patients, with 2 partial responses out of the 9 patients with more than 2 post-baseline scans. In the darovasertib and crizotinib group 1 of 2 evaluable patients had a partial response. IDE196 was found to have synergistic anti-tumor effects when combined with CGM097 (p53-MDM2 inhibitor) or RAD001 (mTORC1 inhibitor) in UM PDX screening studies, suggesting other new potential combination therapies for IDE196 in MUM [140].

Oncogenic Gα signaling also results in constitutive activation of the PI3K/AKT/mTOR pathway. A small 13 patient phase II trial of the mTOR inhibitor everolimus combined with somatostatin receptor agonist pasireotide had limited clinical benefit [141]. Pre-clinical studies suggested PI3K inhibitors had limited effects as monotherapy, but they had synergistic effects against tumor growth when combined with a MEK inhibitor or PKC inhibitor [142,143]. The PKC inhibitor AEB071 and the PI3K-α inhibitor BYL719 drug combination is currently being tested in a clinical phase I study (NCT02273219). The potent synergy between everolimus (mTOR inhibitor) and the PI3K inhibitor GDC0941 was demonstrated in drug combination screening of UM cell lines, which may be another possible combination for MUM [144].

The multi-kinase inhibitor sorafenib, which targets the RAF/MEK/ERK pathway and VEGFR/PDGFR, resulted in limited overall efficacy in a phase II study by the Southwest Oncology Group (SWOG) cooperative group when combined with carboplatin and paclitaxel [145]. In a different phase II study, first-line sorafenib was compared to placebo in MUM patients (STREAM); improvement in median PFS was seen (5.5 months vs. 1.9 months) but there was no OS benefit [146]. Another multi-kinase inhibitor cabozantinib was compared against dacarbazine or temozolomide and no improvement in PFS was seen (NCT01835145) [147]. 

Finally, studies targeting the Hippo/YAP pathway are underway. The non-canonical Gαq signaling pathway activates a highly conserved Rho-GEF, TRIO, and the consequent stimulation of Rho-regulated pathways leading to the activation of YAP, independently of PLC-β [148]. The tyrosine kinase FAK provides a direct link between Gαq and tyrosine phosphorylation networks controlling YAP and has been shown to promote UM growth [149]. FAK is associated with drug resistance as it can be activated in cancer cells when it is exposed to other tyrosine kinase inhibitors. FAK can also be activated by cellular stress and signal through critical nodes in signal transduction pathways such as MEK [150]. It has been shown that targeting FAK kinase activity has the potential to modulate intra- tumoral regulatory T cells (Treg) levels establishing an immunosuppressive tumor microenvironment [151]. Interestingly, UM represents human cancer harboring the highest level of FAK overexpression [149]. Furthermore, *PTK2* expression (the gene that encodes for FAK) has been significantly correlated with reduced overall survival in UM patients, aligning with its potential biological role in UM [150]. UM cell lines demonstrated dose-dependent sensitivity to FAK inhibition by the two FAK inhibitors PF562771 and VS-4718 [149]. VS-4718 treated mice also experienced reduction in UM tumor size [149]. Kinome-wide CRISPR-Cas9 sgRNA screen to identify synthetic lethal gene interactions revealed that FAK and MEK-ERK co-targeting has remarkable synergistic growth-inhibitory effects in UM cells and exerted cytotoxic effects leading to tumor regression in UM xenograft and liver MUM models in vivo [152]. Both the phase II trial of defactinib (VS-6063, FAK inhibitor) and VS-6766 (dual RAF/MEK inhibitor) (NCT04720417) and the phase Ib trial of IN10018 (FAK inhibitor) alone and in combination with cobimetinib (MEK inhibitor) (NCT04109456) are actively recruiting.

### 5.2. EIF1AX

*EIF1AX* mutations have been found to cooperate with *RAS* mutations to induce tumorigenesis [70,71]. The C-terminal *EIF1AX-A113splice* mutation drives an ATF4-induced dephosphorylation of EIF2α, resulting in increased protein synthesis. ATF4 also cooperates with c-MYC, which is stabilized by RAS, to sensitize mTOR to amino acid supply. These mutually reinforcing events may result in vulnerabilities to MEK, BRD4, and mTOR kinase inhibitors [71]. Since metastatic UM rarely possesses *EIF1AX* mutation, *EIF1AX* has not been a target for the treatment of metastatic UM.

### 5.3. SF3B1 and SRSF2

Since *SF3B1* and *SRSF2* are involved in splicing, there has been growing interest in splicing modulators. Several bacterial and synthetic splicing inhibitors have been studied as *SF3B1* inhibitors, including FR901464, spliceostatin B, spliceostatin E, thailanstatin, meyamycin, sudemycin, pladienolides A-G, E7107, FD-895, herboxidiene, and isogingketin [62]. These inhibitors confer high cytotoxic effects by regulating Mcl-1 splicing and inhibiting cell proliferation in a dose-dependent manner [62]. E7107, a semisynthetic derivative of the natural product pladienolide B, was evaluated in a phase I study in patients with advanced solid tumors [153]. Disappointingly no complete or partial responses were observed during treatment. 

*SF3B1* mutations may also be sensitive to nonsense-mediated mRNA decay (NMD) inhibitors [154]. *SF3B1* mutated chronic lymphocytic leukemia, UM, and pancreatic cell lines treated with an NMD inhibitor resulted in increased expression of *SF3B1*-associated cryptic transcripts, suggesting a role of NMD in the pathogenic effects of *SF3B1* mutations [154].

H3B-8800 may be promising as cells harboring *SF3B1* mutations are more sensitive to this inhibitor; in *SF3B1* mutant cells preferential inhibition results in alternative 3′ss enrichment [155]. A phase I, first-in-human study of H3B-8800 in patients with MDS, AML, or chronic myelomonocytic leukemia is ongoing (NCT02841540).

PRMT5 (protein arginine methyltransferase 5) inhibitors are being investigated in *SF3B1* and *SRSF2* mutant tumors including uveal melanoma. PRMT5 directly methylates arginine residues of several splicing factors which contributes to spliceosome assembly and promotes canonical splicing of many essential genes in cancer cells [156]. PRMT5 inhibitors led to increased survival in mice with leukemias with *SRSF2* mutations but not wildtype [157]. Specifically in uveal melanoma, PRMT5 inhibition has been demonstrated to regulate DNA replication and repair pathways [156]. In *SF3B1* mutated UM cells, combining PRT543 (PRMT5 inhibitor) with a PARP inhibitor or DNA-alkylating agent yielded a synergistic reduction in cell viability [156]. There are currently five phase 1 open label, dose-escalation studies of PRMT5 inhibitors in advanced solid tumors: PF-06939999 (NCT03854227), JNJ-64619178 (NCT03573310), GSK3326595 (NCT02783300), PRT543 (NCT03886831), and PRT811 (NCT04089449).

A recent multicenter retrospective analysis evaluated patients with *SF3B1* mutations after immune checkpoint inhibitors [158]. 58 patients with deleterious *SF3B1* mutations received immunotherapy: single-agent PD-1 in 15 patients, single-agent CTLA-4 in 4 patients, and dual checkpoint inhibitor in 15 patients. The median time from initial diagnosis to metastasis was 6.1 years and 29% of patients had non-liver metastasis. 27% had received prior systemic therapy and 35% had received prior hepatic regional therapy. Median OS and 1-year survival after immunotherapy were numerically superior to historical controls. Median OS for all patients from the time of metastasis was 3.9 years, and 1-year OS from time of metastasis was 94% [158]. Response rates were comparable to other immunotherapy trials, with 9% PR and 39% SD [14,23,159]. Bigot et al. demonstrated that *SF3B1* mutations generate shared neoantigens that are uniquely expressed by UM cells, leading to recognition and killing by specific CD8 T cells [160]. This could potentially explain the prolonged survival with immunotherapy and in general for *SF3B1* mutated patients. Future clinical trials may need to stratify for *SF3B1* mutations given the more indolent course of these tumors [158].

### 5.4. BAP1

Binding of BAP1 to ASXL1 results in the formation of the polycomb complex that deubiquinates histone 2A [161]. *BAP1* loss may result in sensitivity to HDAC inhibitors through increased ubiquinated expression. In preclinical UM models HDAC inhibition has been shown to suppress migration and invasion of UM cells, and induce apoptosis, morphologic differentiation, cell cycle arrest, and shift from a high-risk (class 2) to a low-risk (class 1) gene expression profile [162,163]. Many HDAC inhibitors are being studied in UM including valproic acid, panobinostat, vorinostat, tricostatin A, tenovin-6, depsipeptide, MS-275, quisinostat, JSL-1, MC1568, and MCI1575 [62,121,164]. The phase II trial evaluating the HDAC inhibitor vorinostat in MUM patients is recruiting (NCT01587352). As mentioned above, there is an ongoing phase II study of valproic acid with sunitinib in the adjuvant setting for high-risk UM patients (NCT02068586). Six months of adjuvant valproic acid showed a similar 2-year OS rate and 18-month RFS rate as adjuvant sunitinib [116].

The HDAC inhibitor entinostat was combined with the PD-1 inhibitor pembrolizumab in a multicenter, open label phase II study of MUM patients (PEMDAC, NCT02697630) [165]. As of June 21, 2019, the ORR for the 29 enrolled MUM patients was 10% and median OS was 11.5 months [166]. Grade 3 to 4 adverse events were reported in 62% of patients.

The combination of neratinib (HER-2 and epidermal growth factor receptor inhibitor) and entinostat (HDAC inhibitor) resulted in additive cytotoxic effects in PDX of UM, through multifactorial killing via mitochondrial dysfunction and toxic autophagy [167]. MEK inhibition with HDAC inhibition may also hold promise, as the combination resulted in a considerable reduction in growth of both subcutaneous and liver metastasis in xenograft models [168].

*BAP1* loss also leads to increased histone H3 lysine 27 (H3K27), which in turn, increases the expression of EZH2 [169]. However, EZH2 inhibitors did not inhibit tumor growth of UM cells regardless of their *BAP1* status [170]. 

Bromodomain and extra-terminal (BET) inhibitors have been investigated in UM. The BET family of proteins (BRD2, BRD3, BRD4, BRDT) are chromatin readers that bind to acetylated lysine residues on histone tails [171]. BAP1 has been shown to be bridged to BRD4 by a physical interaction between additional sex combs-like protein 3 (AXL3) [172]. BET inhibitors like JQ1 competitively displace BRD4 from acetylated histones, resulting in suppression of c-Myc and c-Myc dependent target genes. Located on 8q24.1, the oncogene *MYC* is amplified in nearly 40% of UM [173]. The BET inhibitor JQ1 had cytotoxic activity in UM cell lines with *GNAQ*/*GNA11* mutations, while in cells without mutations there was little effect [174]. However, when a second-generation BET inhibitor (PLX51107) was tested in different advanced cancers including UM, resistance was seen in UM patients with liver metastases [175]. Combining PLX51107 with AZD4547 (fibroblast growth factor receptor) showed efficacy in UM cells and may be the basis for a novel combination [175]. PLX2853 is another second generation BET inhibitor that is being investigated in advanced malignancies including uveal melanoma (NCT03297424).

The BRG/Brahma-associated factors (BAF) family of chromatin remodeling complexes, also referred to as mSWI/SNF complex, has been found to be a major regulator of lineage- and disease-specific transcriptional programs [176]. Copy number deletions have been found in many subunits of the SWI/SNF complex in *BAP1* deleted malignant mesothelioma tumors, making BAF inhibitors a potential target in *BAP1* mutated tumors [177]. FHT-1015 is a selective inhibitor of SMARCA4 and SMARCA2 (also known as BRG1 and BRM), the ATPase component of the BAF complex [159]. In UM, FHT-1015 resulted in enhancer occupancy reduction of SOX10 and MITF transcription factors, two essential proteins in supporting the proliferation and survival of UM cells. In mouse xenograft models of UM, BAF inhibition was well tolerated and resulted in dose-dependent tumor regression that correlated with pharmacodynamic modulation of BAF-targeted gene expression [159]. A phase I dose escalation and expansion study of FHD-286 in MUM is recruiting (NCT04879017).

Lastly, PARP inhibitors are currently being studied as UM therapy, since *BAP1* deficiency results in impaired homologous recombination. BAP1 forms a complex with BRCA1 and BARD1, forming an E3 ubiquitin ligase that regulates the homologous recombination pathway [178]. Due to impaired homologous recombination, tumor cells are more dependent on other DNA repair pathways. In one study, *BAP-1* loss was found to sensitize renal cancer cells to the PARP inhibitor Olaparib as well as radiation [179]. Loss of *BAP1* in the mouse melanoma cell line B16F10 resulted in increased sensitivity to radiation and rucaparib [180]. A clinical trial of the PARP inhibitor (Niraparib) in BAP1-deficient cancers (UM included) is ongoing (NCT03207347).

## 6. Liquid Biopsy

Genetic and molecular testing of uveal melanoma at this time relies on tissue from enucleation or samples from a fine needle aspiration biopsy (FNAB) of the eye. There is some concern about intratumor genetic heterogeneity and single site sampling resulting in discordance [181]. Liquid biopsy has been gaining popularity in other tumors as a non-invasive alternative approach to analyze molecular features of a tumor. Circulating tumor cells (CTCs), circulating tumor DNA (ctDNA), cell-free microRNA (miRNAs), and tumor-derived extracellular vesicles (EV) are different components that can be analyzed in a liquid biopsy. UM is unique in that it spreads via the blood rather than the lymphatic system, which may lend itself better to a liquid biopsy. Liquid biopsies of the aqueous humor are also under investigation in UM [182]. Liquid biopsies could be a valuable tool for early diagnosis, recurrence, and response to treatment. 

Jin and Burnier recently reviewed the current evidence of liquid biopsies in UM [182]. Advances in technology have resulted in significant progress in liquid biopsies in UM. However, the authors concluded that at this time designing a clinically useful liquid biopsy-based screening test with high enough specificity and sensitivity remains an obstacle. Below we review the different components of liquid biopsies studied so far in UM.

In 1993, Tobal et al. published the earliest report of CTCs in UM [183]. RNA transcripts of melanoma-associated proteins were measured with RT-PCR, and they were able to detect as few as 10 CTCs in 5 mL of blood in 3 of 6 UM patients [183]. Since then, different methods of CTC detection have been explored. One method is using CellSearch which is an FDA-approved CTC detection approach [184]. Using immunomagnetic isolation, cells are first enriched for CD146. Then expression of the high-molecular weight melanoma-associated antigen and staining for CD45 and CD34 allows UM CTCs to be differentiated from leukocytes and endothelial cells. In a study by Bande et al., 50% of primary non-metastatic UM patients had more than 1 CTC detected in their blood sample using CellSearch compared to none in patients with a nevus [185]. In 40 patients with MUM, only 30% had detectable CTCs using CellSearch; those who had detectable CTCs had worse overall survival [184]. CTC positivity and patient outcome was also negatively correlated in another study with 40 UM patients (20 non-metastatic, 19 metastatic) using CellSearch [186]. Mazzini et al. used a detection method based on size filtration and found CTCs in 17 of 31 UM patients, but none in choroidal nevus patients [187]. Significantly worse disease-free and overall survival was found in patients with more than 10 CTCs per 10 mL blood in this study [187]. Cajello et al. reported detectable CTCs in 29 of their 30 UM patients (87.5%) using RT-PCR [188]. CTCs were found in nearly diagnosed, irradiated, enucleated, and observed patients regardless of tumor size and time period following treatment. Of note none of the patients developed metastasis [188]. The CTC detection rates are highly variable across different studies and methods which is problematic [182]. Different methods that may improve detection efficiency include arterial sampling and combining two antibodies instead of a single one [189,190]. 

ctDNA are small fragments of DNA that are released by tumor cells into the blood, likely from apoptosis, necrosis, and active release mechanisms [182]. Bidard et al. used a mutation-specific bidirectional pyrophosphorolysis-activated polymerization (Bi-PAP) technique and found point mutations in *GNAQ* and *GNA11* in the ctDNA of 22 of 26 patients [184]. There was a correlation between ctDNA levels and hepatic miliary metastases, tumor volume, and CTC count. They also found ctDNA to be an independent prognostic factor for PFS and OS. Another studying using the same Bi-PAP technique detected ctDNA in 20 of 21 MUM patients and levels of ctDNA correlated with tumor mass [191]. Metz et al. used an ultradeep amplicon sequencing technique and found *GNAQ* and *GNA11* mutations in ctDNA of 9 of 22 patients, with no correlation to clinical parameters [192]. Jin and Burnier’s group used a different technique of digital droplet PCR for ctDNA detection of *GNAQ*, *GNA11*, *PLCB4*, and *CYSTLR2* mutations in 40 patients with UM, nevus, and healthy subjects, and found a strong correlation between ctDNA detection and disease [182]. 

Recently, the ctDNA of 17 MUM patients treated with a PKC inhibitor in a phase 1 clinical trial were analyzed [193]. This study found baseline ctDNA to be strongly correlated with baseline lactate dehydrogenase level and baseline disease burden. ctDNA accurately predicted patients with clinical benefit to PKC inhibitor and detecting disease progression prior to radiological progression [193]. ctDNA use in the phase 2 IMCgp100-102 trial of tebentafusp (IMCgp100) in previously treated MUM patients was also recently presented at the 2021 European Society for Medical Oncology (ESMO) Congress [194]. Reduction of ctDNA by Week 9 while on tebentafusp was strongly associated with OS. 14% of patients had complete ctDNA clearance and long OS, including some patients with the best response of stable or progressive disease. The majority (70%) of evaluable patients had any ctDNA reduction while 5% of patients had radiographic response per the RECISTv1.1 criteria, suggesting ctDNA may be more sensitive than RECIST [194].

miRNAs are small noncoding RNAs that function as regulators of gene expression that can impact cancer development [182]. Several studies have found circulating miRNAs differentially expressed in UM patients compared to healthy controls [195,196,197,198,199,200]. The circulating miRNAs differ between the studies, but circulating miR-164a seems to be consistently found in UM patients with further increase upon the development of metastasis [196]. 

Extracellular vesicles are nanoparticles that are categorized mainly into microvesicles, apoptotic bodies, and exosomes [182]. RNA, DNA, and proteins are transported by EV. Studies on EV in UM are limited. Eldh et al. isolated exosomes from liver perfusate of 12 UM patients with liver metastases and found a higher concentration of exosomes in systemic blood in those patients compared to healthy controls [201]. The exosomes of the UM patients also contained a similar miRNA profile between patients, but dissimilar to other tumor cell lines [201].

## 7. Conclusions

The genetic landscape of uveal melanoma is unique when compared to other tumors. UM tumors have a low tumor mutational burden, but they do have specific recurrent mutations. UM tumors often harbor a tumor-initiating mutation in *GNAQ* or *GNA11* with secondary mutation in *EIF1AX*, *SF3B1*, *SRSF2*, or *BAP1* that determines metastatic potential. UM tumors are also characterized by distinct chromosomal abnormalities that are tightly linked to prognosis; in particular, monosomy 3, gain of 8q, and loss of 8p are associated with higher metastatic risk. GEP also adds to the prognostication of primary UM tumors, all of which aids clinicians and their patients in determining surveillance patterns and the possibility of adjuvant therapy trial enrollment. In addition to prognosis, UM-specific mutations have become valuable targets in a landscape where there are few effective therapies for MUM. Finally, there is some variability in survival outcomes in patients with MUM, which may be explained by differences in molecular and cytogenetic mutations of the tumor; monosomy 3 tumors appear to have worse outcomes and *SF3B1* mutated tumors with improved outcomes, but this needs to be elucidated further [16,158,202].

Most of our mutational knowledge is based on primary UM and a better understanding of the genetic landscape in metastatic UM is needed. Liquid biopsies, especially ctDNA, focusing on UM-specific mutations are promising and could become an important biomarker for future clinical use. 

In summary, primary tumor biopsies should be performed to obtain genetic information of UM as it provides valuable insight into a patient’s prognosis, surveillance, and treatment options.

## Figures and Tables

**Table 1:** Ongoing Adjuvant Therapy Trials.

Trial Drug(s) and Mechanism of Action	Phase	Identifier	Status
Sunitinib (KIT inhibitor) + Valproic Acid (HDAC inhibitor)	II	NCT02068586	Recruiting
Crizotinib (MET inhibitor)	II	NCT02223819	Active, Not recruiting
Dendritic cell vaccination (immunotherapy)	III	NCT01983748	Recruiting
Nivolumab (PD1 inhibitor) +/− Ipilimumab (CTLA4 inhibitor) or Relatlimab (LAG3 inhibitor)	II	NCT02519322	Active, Not recruiting
Ipilimumab (CTLA4 inhibitor) + Nivolumab (PD1 inhibitor)	II	NCT03528408	Recruiting

**Table 2:** Ongoing Targeted Therapy Trials for MUM.

Trial Drug(s) and Mechanism of Action	Phase	Identifier	Status
BVD-523 (ERK1/2 inhibitor)	II	NCT03417739	Active, Not recruiting
Intermittent Selumetinib (MEK inhibitor)	IB	NCT02768766	Recruiting
LXS196 (PKC inhibitor) +/− HDM201 (MDM2 inhibitor)	I	NCT02601378	Active, Not recruiting
IDE196 (PKC inhibitor) +/− Binimetinib (MEK inhibitor) or Crizotinib (MET inhibitor)	I/II	NCT03947385	Recruiting
AEB071 (PKC inhibitor) + BYL719 (PI3K-α inhibitor)	I	NCT02273219	Active, Not recruiting
Defactinib (FAK inhibitor) + VS-6766 (RAF/MEK inhibitor)	II	NCT04720417	Recruiting
IN10018 (FAK inhibitor) +/− Cobimetinib (MEK inhibitor)	IB	NCT04109456	Recruiting
Cabozantinib (multi-kinase inhibitor) vs. Dacarbazine (alkylating agent) or Temozolomide (alkylating agent)	II	NCT01835145	Active, Not recruiting
H3B-8800 (SF3B complex modulator)	I	NCT02841540	Recruiting
PF-06939999 (PRMT5 inhibitor)	I	NCT03854227	Recruiting
JNJ-64619178 (PRMT5 inhibitor)	I	NCT03573310	Active, Not recruiting
GSK3326595 (PRMT5 inhibitor) +/− Pembrolizumab (PD1 inhibitor)	I	NCT02783300	Recruiting
PRT543 (PRMT5 inhibitor)	I	NCT03886831	Recruiting
PRT811 (PRMT5 inhibitor)	I	NCT04089449	Recruiting
Vorinostat (HDAC inhibitor)	II	NCT01587352	Recruiting
Entinostat (HDAC inhibitor) + Pembrolizumab (PD1 inhibitor)	II	NCT02697630	Active, Not recruiting
PLX2853 (BET inhibitor)	IB/IIA	NCT03297424	Recruiting
FHD-286 (BAF inhibitor)	I	NCT04879017	Recruiting
Niraparib (PARP inhibitor)	II	NCT03207347	Recruiting
